# Quantifying finer-scale behaviours using self-organising maps (SOMs) to link accelerometery signatures with behavioural patterns in free-roaming terrestrial animals

**DOI:** 10.1038/s41598-021-92896-4

**Published:** 2021-06-30

**Authors:** Nicole Galea, Fern Murphy, Joshua L. Gaschk, David S. Schoeman, Christofer J. Clemente

**Affiliations:** 1 School of Science and Engineering, University of the Sunshine Coast, 90 Sippy Downs Drive, Sippy Downs, QLD 4556 Australia; 2 Global-Change Ecology Research Group, University of the Sunshine Coast, 90 Sippy Downs Drive, Sippy Downs, QLD 4556 Australia; 3 Department of Zoology, Centre for African Conservation Ecology, Nelson Mandela University, Port Elizabeth, South Africa

**Keywords:** Behavioural methods, Behavioural ecology, Animal behaviour, Invasive species, Urban ecology

## Abstract

Collecting quantitative information on animal behaviours is difficult, especially from cryptic species or species that alter natural behaviours under observation. Using harness-mounted tri-axial accelerometers free-roaming domestic cats (*Felis Catus*) we developed a methodology that can precisely classify finer-scale behaviours. We further tested the effect of a prey–protector device designed to reduce prey capture. We aligned accelerometer traces collected at 50 Hz with video files (60 fps) and labelled 12 individual behaviours, then trained a supervised machine-learning algorithm using Kohonen super self-organising maps (SOM). The SOM was able to predict individual behaviours with a ~ 99.6% overall accuracy, which was slightly better than for random forest estimates using the same dataset (98.9%). There was a significant effect of sample size, with precision and sensitivity decreasing rapidly below 2000 1-s observations. We were also able to detect a behaviour specific reduction in the predictability when cats were fitted with the prey–protector device indicating it altered biomechanical gait. Our results can be applied in movement ecology, zoology and conservation, where habitat specific movement performance between predators or prey may be critical to managing species of conservation significance, or in veterinary and agricultural fields, where early detection of movement pathologies can improve animal welfare.

## Introduction

Bio logging devices, in particular tri-axial accelerometers, can be used to investigate finer-scale behaviours and the biomechanical patterns of movement which are relevant to species conservation planning (Houghton et al.)^1,2^. However, research using accelerometry to identify the finer-scale behaviours associated with individual activities of a species is currently scarce^3^. Fine scale behaviours can be characterised as biomechanic movements of short duration (eg. pouncing and jumping). Behaviours measured with accelerometers quickly produce millions of data points, making analysis of acceleration data a time-consuming task. This is one of the main challenges when using this technology for the study of animal behaviour^4,5^. Despite the improvements in technology to understand animal movements, an easy-to-use, effective tool to process and identify finer-scale behavioural patterns from accelerometer data is currently lacking.

Increasing attention has focused on automated behavioural classification using machine-learning (ML) techniques to classify the behaviour of animals from accelerometry^6^. Machine learning techniques such as support vector machines (SVMs), random forest (RF) and artificial neural networks (ANNs), provide computationally powerful methods of data classification that can detect complex patterns that are not evident to the human eye. Thus, the methods can identify intrinsic differences between behaviours or locomotory types when applied to acceleration data^7,8^. Accelerometer data have been used to compare machine learning methods in a study by Nathan et al.^9^. Supervised methods (ANN, RF, SVM) were implemented and compared with linear discrimination analysis (LDA) as a baseline for classifying seven behavioural modes in free ranging griffon vultures. All machine learning methods were found to accurately classify behaviour (80–90%) and all non-linear methods outperformed LDA.

Self-organizing maps or (SOMs) are a type of ANN which can efficiently create maps of multi-dimensional and complex data in order to approximate the probability density function of the input data and show the data in a more comprehensive fashion and in fewer dimensions^10,11^. SOMs have been implemented broadly in ecological sciences, the methodology having advantages for information extraction (i.e., without prior knowledge) and efficiency of presentation, (i.e., visualization)^10^. The algorithm is able to map high-dimensional data to a 2-dimensional map display in such a way that similar data are located close to each other on the map^12^; allowing simple interpretation of large data sets. Use of SOMs in ecological behavioural literature have primarily analysed behavioural change in animals in response to environmental stressors (e.g., toxic chemicals)^10,13–15^. The method was also recently applied to classify behaviour in the human gait signature. Lakany^16^ successfully used wavelet and SOMs to correctly classify pathological cases which clinicians, due to the complexity of the impairment, have difficulty in accurately diagnosing. The SOM extracted features that successfully discriminated between those individuals with and without impaired locomotion^10,16^.

There is limited research in animal behaviour that uses the practice of pairing the trained finer-scale individual movements from accelerometer trace data sets and applies it to trained supervised (ML) SOM algorithms, to produce higher levels of accuracy in detecting how different species are behaving on the basis of independent unsupervised data sets^3,7,17^. Here we demonstrate the use of SOMs to visually represent and compare 12 different behaviours in free roaming domestic cats.

Domestic cats are highly effective hunters, displaying variations in hunting tactics and a range of prey-specific behaviours^18,19^. In order to reduce this impact of domestic cats on wildlife many manufacturers have attempted to create prey protector devices which reduce the effectiveness of hunting in domestic cats. One example is the CatBib which was shown to reduce prey capture by 81%^20^. These studies suggest that the CatBib™ prey protector device could disrupt in some way the killing behaviours of cats^20,21^; however, there is no clear evidence or research on how or why this occurs. Without a mechanistic understanding of how the CatBib™ influences the gait or movements of cats, it is challenging to ascertain whether the CatBib™ causes a reduction in overall hunting behaviours, or simply acts as a visual deterrent. We therefore further demonstrate the effectiveness of the SOM procedure to distinguish not only individual behaviours, but the ability to detect behaviour specific changes in movement which result from the presence or absence of the bib.

We studied the behaviours of free-roaming domestic cats (*Felis catus*) within South East Queensland, Australia, with a particular focus on developing tools to allow fine-scale movement behaviours of small animals to be collected and easily displayed. We used accelerometer trace data to identify behaviours, using a supervised machine learning self-organising map (SOM) algorithm to accurately predict the foraging behaviours of domestic cats^22^. Our objectives were to determine: (1) how accurate SOMs are in predicting fine-scale behaviour and (2) how the CatBib influences behavioural signatures in the accelerometer trace, if at all. It is expected that the accelerometer signature will be modified while wearing the CatBib when cats are free roaming, with hunting behaviours that display intensive acceleratory bursts of short duration such as jumping and pouncing to be most impacted.

## Methods

### Data collection

Data were collected in the Sunshine Coast region in Queensland, Australia (− 26.65° S, 153.07° E), from February to April 2019. All methods were carried out in accordance with relevant guidelines and regulations. All experimental protocols and methods were approved and carried out in compliance with the ARRIVE guidelines under the approval of the University of the Sunshine Coast (USC) Animal Ethics permit (ANA/16/109T); Human Ethics permit (A181114) and in conjunction with the Sunshine Coast Council (SCC) Local Law permit (OM18/19).

### Animals used in the trials

We recruited 10 domestic cats through an approved media release (males n = 6; females n = 4; weight 2.8–8.4 kg; age 1.5–12 years; body length 38–53 cm; foreleg length 16–19 cm). As per the Sunshine Coast Council local law requirements, all cats had to be neutered, registered and microchipped to participate in the study.

### Equipment

We fitted each cat with a retail harness, to which we attached a tri-axial accelerometer (AX3; Axivity, Newcastle University, UK; 23 × 32.5 × 8.9 mm; 11 g) using cable ties (Fig. 1a). The accelerometer was initialised using the Open Movement Graphical User Interaction application (OMGUI; V1.0.0.37). Because a trade-off exists between data resolution and battery life, we logged data at 50 Hz and with a dynamic range of ± 8 g, with a 13-bit resolution, similar to a previous study^23^. When combined with the in-built memory storage capacity of 512 MB, and battery limitations, this configuration resulted in a maximum of 8–14 days of data collection. The quartz Real Time Clock and calendar provided a timestamp with a frequency of 32.768 kHz and a precision of ± 50 ppm, with manufacturer specifications indicating a drift of 0.18 s per hour. To overcome this drift over the eight days, we calibrated devices by video recording the signals of five claps/taps on the device, at the start and end of each individual data collection period, and also at random times during the day.

**Figur﻿e 1:**
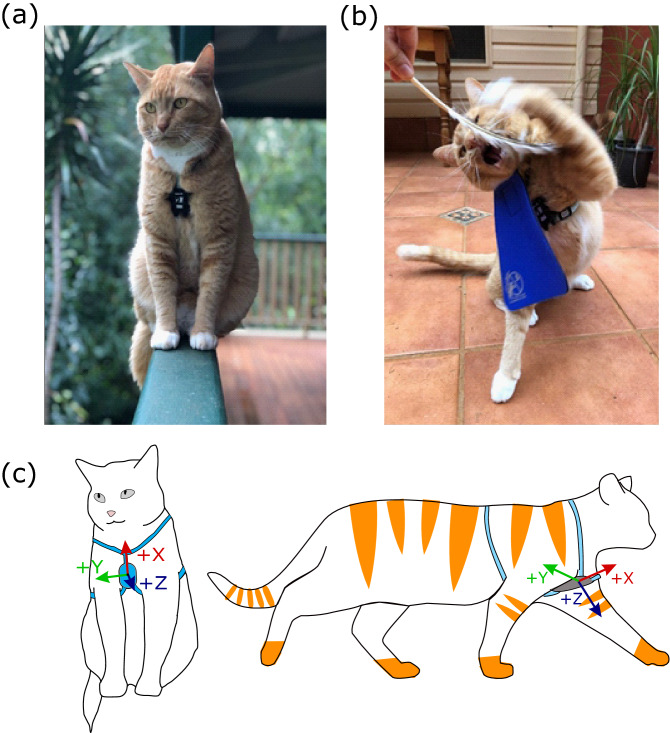
(**a**) The anatomical position of the accelerometer (AX3) on the sternum of the cat. (**b**) The activity of swatting stimulated by the use of a feather. (**c**) The axis orientation of the accelerometer planes, which are represented in the accelerometer trace data in the MATLAB interface. Fore-aft (surge), lateral (sway) and dorso-ventral (heave) movement is reflected in the X, Y and Z signals.

We positioned the accelerometer on the scapular brace-strap of the harness, inverted such that the accelerometer was on the sternum of the cat (Fig. 1a–c). Field trials over four months on four cats in the study determined that this position, in comparison with mounting on the dorsal cranial median plane, did not interfere with the animals’ balance; it also removed all of the abnormal movement behaviours and unnecessary discomfort to the cat^2^. The positioning of the logging device on the frontal anterior, median plane, resulted in the primary axis for fore-aft (surge), lateral (sway) and dorso-ventral (heave) movement to be reflected in the X, Y and Z signals, respectively (Fig. 1c).

The accelerometer harness was used in conjunction with the CatBib for the relevant treatment periods. The total combined mass of the harness, accelerometer and Catbib was to 34.1 g, with a minimum cat mass of 2.8 kg, suggesting the equipment did not weigh above 1.2% of total body weight in any cat studied. The CatBib is a prey protector device, manufactured from a lightweight, washable neoprene material, that is attached to a cat’s safety collar (Fig. 1b). The dimensions of the bib are 17.5 mm × 17.5 mm × 6.5 mm, with a total mass of 23.1 g and it is purple in colour. All cats adjusted to the harness and CatBib within the first hour of deployment and no subsequent adjustments were required. All cats had unrestricted access to roam freely outside during the eight days of field trials.

To capture training data, each cat was filmed with a GoPro + 3 Hero device (H.264—1920 × 1080; f/2.8; 60 fps), undertaking natural or stimulated active behaviours through play (Fig. 1b). These activities or behaviours were manually documented to track the activity, date and the timestamps. We conducted two treatments over the eight days: in the first, cats were fitted with CatBib, whereas in the other, bibs were not worn. Each treatment was conducted for four consecutive days, and the sequence of treatments for each cat was randomised. The accelerometer device on the harness was left on the cats for the entire field trial and recorded continuously for the eight days (~ 192 h per cat; total = 2304 h).

### Data analysis

Each accelerometer trace file was exported as a raw binary file through OMIGUI and imported into a custom-built MATLAB GUI. To build our training dataset, the video file timestamp information, determined using Mediainfo (version 18.08, 2018), was used to define the start time for a subset of the accelerometer trace, and the video length to define the end point (Supp. Fig. 1). Offsets between the accelerometer trace and video files were determined using the closest calibrated tap signal trace for each day. We were able to watch each video file in synchrony with the accelerometer trace, and manually annotate each movement/activity from the video files to the accelerometer subset (Clemente et al.)^24^ (Supp. 1.1. Matlab interface instructions; Supp. Fig. 1).

We grouped activities according to behaviour into three classes: Sedentary, Eating and Locomotive and Hunting. We further subdivided each group into behaviours. Sedentary included lying, sitting, grooming and watching; Eating and Locomotive included—eating/drinking, walking, trotting; and for Hunting—galloping, jumping, pouncing, swatting, biting/holding (Supp. Table 1).

The accelerometer trace was then further divided into rolling epochs of 50 samples in length, using 1 s duration at 50 Hz to ensure intensive acceleratory bursts of short duration such as jumping and pouncing are captured. The behaviour with the maximum frequency within each epoch was assigned as that epoch’s label. Raw accelerometer data in each epoch was assigned as that epoch’s label. Raw accelerometer data in each epoch was summarized using 26 of the most effective variables for procedure accuracy identified by Tatler et al.^25^. We included: axial acceleration (X, Y, Z),mean acceleration (X, Y, Z); minimum acceleration.(X, Y, Z); maximum acceleration (X, Y, Z); standard deviation of acceleration (X, Y, Z); Signal Magnitude Area, minimum Overall Dynamic Body Acceleration (ODBA); maximum ODBA, minimum Vectorial Dynamic Body Acceleration VDBA; maximum VDBA, sum ODBA; sum VDBA; correlation (XY, YZ, XZ); skewness (X, Y, Z); and kurtosis (X, Y, Z)^25^ (See Supp. Table 2 for a detailed description of each variable). Finally, we coded the two treatments: Bib_ON_ and Bib_OFF_ and included this information in the training data set.

### Classification modelling

To determine whether we could predict cat hunting behaviours, we analysed the training data sets using a Kohonen super Self Organising Map (SOM) in the R package ‘Kohonen’ version 2.0.19^26,27^.

Machine learning procedures such as random forest and support vector machines each provide computationally powerful methods of data classification, however each method is not equal in how it visualises its output. SOMS have been used in behavioural studies^10,13–15^ for their ability to efficiently create easily interpreted maps and identify patterns of behaviour. Self-organizing maps differ from other artificial neural networks as they apply competitive learning as opposed to error-correction learning. In this study, a self-organising map algorithm was chosen for its efficiency in visualising multi-dimensional and complex data onto an easily interpreted two dimensional map output. SOMs also have the ability to visualise which variables are most influential with the use of component planes (Fig. 3b–e) and unlike other procedures mentioned, SOMs use cluster analysis which in this study aids in identifying similar behaviours and visualising them closer together (in clusters) on the map output.

To prepare data for the SOM function a random sample of the classifiers for the trained data were extracted, along with their associated behaviour, and combined into a list with 2 elements (measurements and activity). This list was then input into the function supersom.R function, with the grid argument defined using the somgrid.R function [e.g. supersom(TrainingData, grid = somgrid(7, 7, "hexagonal"))]. The 7 × 7 grid function was chosen based on a sensitivity analysis exploring all combinations of grids between 4 to 9 units in length (n = 36, Supp. Fig. 2). The 7 × 7 grid represented the grid which produced the highest accuracy and map symmetry^26,28,29^. We further tested the effect of the number of times the complete data set is presented to the network by varying the rlen argument in the supersom.R function. We found no obvious increase in overall accuracy with increased iterations, and therefore used the default length of 100 times (Supp. Figure 3). Each supersom procedure created was then tested using the predict.R function, with the newdata argument directed to a testing data set, which was a similar 2 element list containing all samples not included in the training data set. The result of this test was then assembled into a confusion matrix using the table.R function with predictions compared with the known behaviours in the test data set [e.g. table(predictions = ssom.pred$predictions$activity, activity = testData$activity) ]. A confusion matrix is a table where each row represents the instances in a predicted class, while each column represents the instances in the observed class, allowing mislabelled epochs to be easily identified. The confusion matrix was then finally used to compute four specific accuracy metrics—sensitivity (or recall), precision, specificity, as well as overall accuracy.

To identify relationships between the size of training dataset, we trained a randomised subset of the Bib_OFF_ training data, to predict the remaining Bib_OFF_ data from all cats. We tested 35 different subset sample sizes from 100 to 100,000, replicating each sample size ten times (with replacement) to determine variation at each sample size.

We then tested the extent to which accelerometer traces are modified by the presence of the CatBib. This modification was indicated by a change in overall prediction accuracy of the SOM between Bib_OFF_ and Bib_ON_ treatments. To do this, we trained the SOM using a subset of the trained data for Bib_OFF_ and tested it against annotated classified Bib_ON_ samples. In order to statistically compare results from bootstrap resampling, we took the median among bootstrap samples as the estimate of performance and quantified uncertainty using the corresponding 2.5th and 97.5th percentiles to represent credible 95% confidence intervals (CIs). We chose the median as a measure of central tendency, because resampling distributions were truncated at 1, so were skewed. If CIs for any pair of estimates (medians) do not overlap, then this is evidence of a significant difference between the estimates. If, however, one estimated median fell within the confidence interval for another estimate, then this was used as evidence of a lack of significant difference. For all other outcomes, differences are equivocal, and we interpreted them tentatively on the basis of the relative overlap in CIs.

Finally we compared the output of the SOM with the output from a decision tree classification method using a random forest (RF) approach from the randomForest.R package^30^. We chose random forest as a comparison as this method has previously been shown to perform better than other similar methods (e.g. k-nearest neighbour, support vector machine, and naïve Bayes) when classifying behavioural data on free moving animals^25,31^. We trained both the SOM and RF procedures using the same 20,000 randomly selected epochs, and compared the overall accuracy for predicting the behaviour for the remaining ~ 192,000 epochs. The SOM was built using a 7 × 7 grid patterns, with the rlen argument set to 100. The RF was built with the number of trees set to 100 and the number of variables randomly sampled as candidates at each split set to 4.

## Results

### Collected data

We collected 2304 h of data from 10 cats over a period of four weeks which included 103.47 h of labelled data from the two CatBib treatments. This resulted in 212,789 1-s epochs of labelled data in the Bib_OFF_ condition, and 159,724 1-s epochs of labelled data in the Bib_ON_ condition. The output resulted in high overall accuracy (99.6%) when a random sample of 20,000 epochs was included (Supp. Table 3–4). Specificity was similarly high (99.8%), with slightly lower levels of precision (93.3%) and sensitivity (89.2%).

### Method performance and behaviour detectability

**Figure 2:**
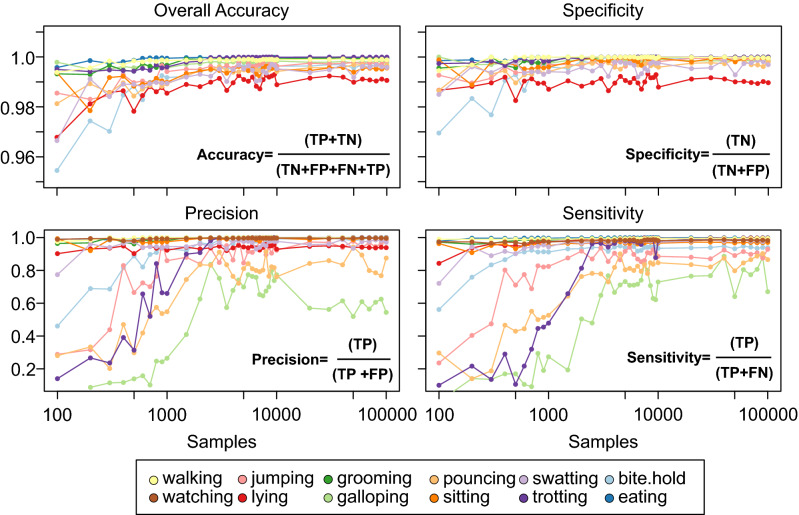
Predictability of the SOM procedure across four behaviour specific metrics. Predicted behaviours were labelled as true positive (TP) if they correctly matched the actual behaviour, true negative (TN) if they correctly identified as a different behaviour, false positive (FP) if the behaviour was incorrectly identified, and false negative (FN) if they incorrectly identified as a different behaviour^25^. These labels were then used to compute four specific accuracy metrics—sensitivity (or recall), precision, specificity, as well as overall accuracy based on the formulas presented in each subplot.

Of the 212 789 samples in the Bib_OFF_ data we took random sub samples between 100 and 100,000 to test how accuracy changes with sample size. The sensitivity of the procedure increased dramatically in behaviour detection for sample sizes between 2000 and 10,000 samples but plateaued after 20,000 samples (Fig. 2). However, lower sample sizes of less common behaviours such as galloping (76%) and pouncing (83%) may have hindered the procedure’s ability to detect overall accuracy of these behaviours (Supp. Table 7). In contrast, activities which were common: lying (99%), walking (99%) and eating (99%) had the greatest accuracy even at very low sample sizes < 1000 epochs long.

**Figure 3:**
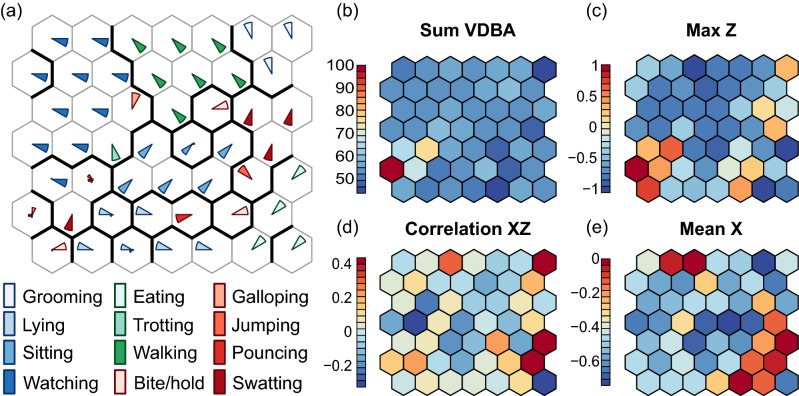
(**a**) The self-organising map comprising 49 hexagonal cells, each identifying a unique accelerometer signature. The thick black lines separate and cluster the identified behaviours in which the procedure believes are most similar. A single triangle represents a behaviour identified 100% of the time. The colour of each triangle indicates which activity is represented in the cell, and proximity of cells to each other indicates the similarity of their signature. (**b**–**e**) Component planes for four example classifiers with heat maps indicating the relative importance of classifiers among different behaviours.

Figure 3a illustrates the SOM output, using the plot.kohonen.R function with the ‘type’ argument set to codes. The colour of each triangle indicates which activity is represented in the cell, and proximity of cells to each other indicates the similarity of their signature. Behaviours which share similarity can be seen clustering together; for example activities which include movement; walking, grooming and swatting in the top right of the map. Sedentary activities lying and sitting are shown clustering to the centre bottom of the SOM output. The bold lines between cells in Fig. 3a are defined using hierarchical clustering using the distances between codebook vectors in the SOM output [e.g. cutree(hclust(object.distances(ssom, "codes")),12) ].

Figure 3b–e illustrates the SOM output using the plot.kohonen.R function with the type argument set to property. This produces component planes for each of the classifiers with heat maps indicating the relative importance of classifiers among different behaviours. Four example component planes are shown. The SumVDBA is shown associated with jumping, Max Z is associated with jumping and biting, the correlation between X and Z was associated with eating and grooming, while the mean X trace was associated with watching, walking, lying and eating. Fore-aft (surge), lateral (sway) and dorso-ventral (heave) movement is reflected in the X, Y and Z signals.

### Biomechanical effects of the CatBib

**Figure 4:**
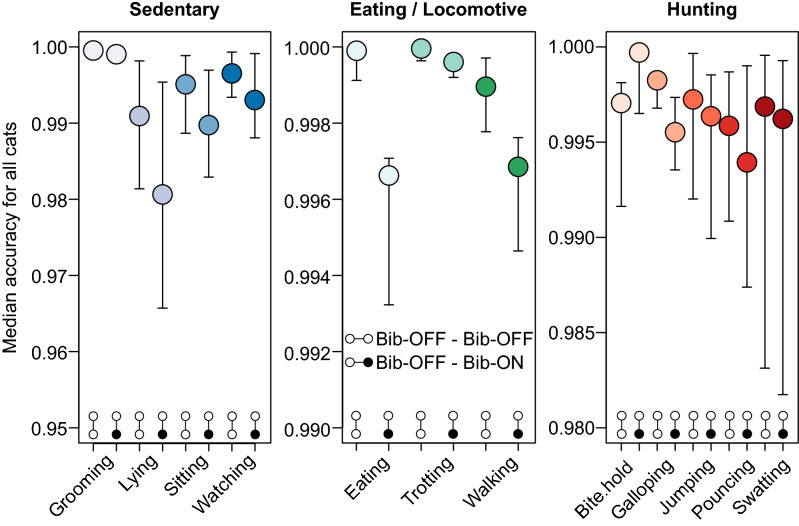
The prediction accuracy of the SOM trained with a random sample of 20,000 1-s epochs of Bib_OFF_ data for all cats to predict either Bib_OFF_ (n = 231,570) or Bib_ON_ samples (n = 175,989), separated by activity. Colours represent grouped behaviours, with sedentary activities represented by blue colours, eating and locomotive by green and hunting activities by red. Within each activity, we interpret changes in predictability as modifications in the accelerometer signature in response to the cat bib.

For further analysis we used a random subsample of 20,000 1-s epochs, which reflected a compromise between accuracy and computational time. Using the trained subset of Bib_OFF_ data, we tested whether the bib had any biomechanical effect on the labelled Bib_ON_ data set (i.e., whether the predictability of activities declined for the Bib_On_ relative to the the Bib_Off_ treatment). This analysis was repeated 1000 times for Bib_OFF_ versus Bib_OFF_ data, and similarly for Bib_OFF_ versus Bib_ON_. Non overlapping CI’s indicate the biomechanics in activities eating and walking were affected by the presence of the bib. We detected a reduction in predictability when the SOM was trained using Bib_OFF_ data and tested on the Bib_ON_ condition. Further, this reduction in predictability was inconsistent among behaviours, allowing us to determine which behaviours were most influenced by the presence of the Bib_ON_ (Fig. 4). Of these behaviours, eating and walking had the overall largest effect size when all of the cats were considered.

### Comparison with random forest procedures

**Figure 5:**
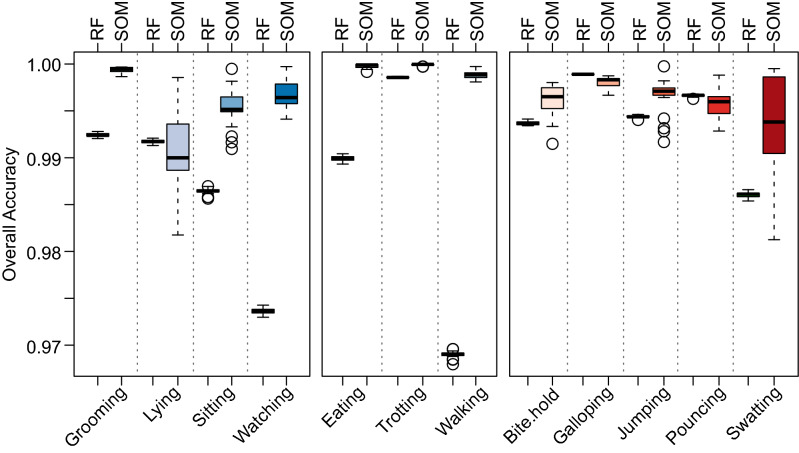
A comparison of the self-organising maps (SOM) procedure with a random forest (RF) classification procedure separated by behaviour. Each procedure was trained with the same random sample of 20,000 1-s epochs of Bib_OFF_ data for all cats to predict the remaining Bib_OFF_ (n = 231,570) samples. Colours represent grouped behaviours, with sedentary activities represented by blue colours, eating and locomotive by green and hunting activities by red.

When all behaviours were combined the SOM procedure appeared to outperform the RF procedure based on three of the four specific accuracy metrics. The SOM procedure displayed a higher overall accuracy, precision and specificity, but the RF procedure generally displayed better sensitivity (Supp. Tables 3–6). The difference in the overall accuracy between the two methods was also dependent on the behaviour. Figure 5 shows the relative accuracy of each method to predict various behaviour; the SOM procedure outperformed the RF procedures in 9 of the 12 behaviours (based on non-overlapping confidence intervals). The RF procedure performed better than the SOM for galloping and pouncing, which were both represented by low sample sizes (Supp. Table 7). Both procedures performed equally well at predicting the lying behaviour, though the variance in accuracy was greater for the SOM procedure (Fig. 5). The average computational time for the SOM procedure was 7.55 s, while the computational time for the RF procedure was 13.56 s.

## Discussion

This paper developed a method to accurately identify finer-scale behaviours from accelerometer trace signatures. Few studies have been able to identify fine-scale behaviours with high accuracy^2–4,17,25,32,33^. Our objectives were to determine how accurate SOMs are for predicting fine-scale behaviours from accelerometer signatures in free-roaming domestic cats and whether these signatures are influenced by the CatBib prey protection device.

We compared the accuracy of SOMs with other studies that used comparable classification procedures. Carrol et al.^7^, applied accelerometer data to a support vector machine (SVM) to identify a prey capture signature for little penguins, the study showed that fine scale foraging behaviours that correspond to transient events lasting less than a second can be detected with a machine learning procedure such as an SVM with an accuracy of 84.95 ± 0.26% (mean ± s.e.). Research by Fehlmann et al.^34^ uses a random forest procedure (RF) to analyse fine scale behaviours in foraging and locomotion on primates using tri axial accelerometer data. The machine learning analysis identified all behaviours with an average precision of 88.3% (± 8.5%) and a mean recall of 70.7% (± 29.3%) across all behaviours. Tatler et al.^25^ used a cross-validation method comparing performance of four commonly used classification procedures (Naïve Bayes, SVM, *k-*nearest neighbor & RF) at a sampling frequency of 1 Hz to identify 14 behaviours observed from three captive dingos (*Canis dingo*). The True skill statistic score was highest with overall accuracy 87% when using a random forest procedure.

The SOM delivered a consistently high (~ 99%) accuracy from free-roaming domestic cats across all behaviours at a sampling frequency of 50 Hz, at least where sample sizes are greater than ~ 2000. This appeared to perform as well or better than a random forest using an identical dataset (Fig. 5; Supp. Tables 3–6). The SOM has an added advantage over other machine learning procedures, in its ability to easily create heat mapped component planes (Fig. 3b–e) which help to determine the contribution of each variable to cluster structures and the correlation between the different variables in the dataset (Pacella et al.)^35^. Visualising which variables are colinear is useful in determining which to include in the procedure, and which variables have the greatest explanation in the output. The clustering capability of similar behaviours is also a strong visual advantage provided by the SOM. The dissimilarity in behaviours can be seen between watching and eating which are clustered furthest apart in the top left hand corner and bottom right hand corner. Closer together we can see grooming and swatting sharing the top right hand corner, indicating these behaviours are more alike. Watching and walking both have large representation from the dataset on the map and do not appear clustered within other behaviours. With our dataset containing a larger amount of these behaviour data points, the SOM has efficiently recognised these behaviours by clustering them strongly together. However, limitations of the SOM network are illustrated in the bottom left hand corner. The behaviours with low sample sizes are represented by mixed triangles that indicates the SOM network was unable to consistently identify the behaviour with high precision or sensitivity. These behaviours included pouncing (n = 3884) and galloping (n = 715), which sample sizes were significantly lower than other behaviours (Supp. Table 7). Sufficient data is required in order to develop strong clusters and give accurate precision, and in these cases the RF approach may yield better results (Fig. 5). Where sample sizes were low in our data set, precision and sensitivity were weakest and more likely to produce a false positive or false negative output (Fig. 2). Future studies should focus training data collection towards relatively rarer behaviours that are associated with hunting and foraging activities such as in our data set galloping and pouncing.

We were able to detect a reduction in predictability when the SOM was trained using Bib_OFF_ data and tested on the Bib_ON_ condition. This suggests that the CatBib is interfering with these behaviours and, therefore, cats have had to adjust their biomechanical movement of these activities. This effect was not unexpected and is likely due to the proximity of the cat bib to the anterior limbs. Walking is likely affected by a physical interaction of the limbs with the bib as they swing through each stride. Similarly, eating is likely to be greatly affected by the bib interacting with the head when the cat adopts a sitting posture during feeding and the bib hangs in front of, or even over the food. Yet there was little evidence that activities associated with hunting behaviours, including galloping, swatting and pouncing, were influenced by the bib (Fig. 4). This may reflect the low predictability of these activities. Alternatively, the accuracy of these behaviours may not have been greatly altered as these are powered by the hind feet, and therefore less likely to be affected by the presence of the bib. If the latter is true, then this may suggest the reduced kill rate while wearing the bib can be primarily attributed to increased visual exposure of the cat, rather than biomechanical alterations to the gait. It must also be considered, given our results, the reductions in kill returns reported by Calver et al.^20,36^, might be because of interference with bite/hold activity, as cats may be able to kill, but not carry prey. Such a possibility requires further substantive investigation.

The SOM demonstrates a highly accurate (~ 99%) procedure when classifying fine-scale behaviours from a small (< 8 kg) terrestrial predator. We show that the self-organising map algorithm performs well if not better than other procedures such as random forest when overall accuracy is compared. The advantages of SOMs have been demonstrated through visualisation of output, of both the map and its respective component planes, and the strength in its ability to determine weights of individual classifiers. Finally, the SOM has shown its ability to detect behaviour specific changes in response to the CatBib prey protector device. SOMs are highly underrepresented in behavioural classification despite the strengths of the approach. These features combined with relative ease of implementation make SOMs highly suitable for work in ecology and this study provides a template for future studies in ecological feature detection and classification.

## Supplementary information

Supplementary Informations.

## Data Availability

Graphical Matlab interactive interface code and training notes: https://figshare.com/articles/Galea_et_al_2019/9978797; R Code: https://figshare.com/articles/Galea_et_al_2019/9978797; Data: https://figshare.com/articles/Galea_et_al_2019/9978797.
