# Supplementary material for: Quantifying finer-scale behaviours using self-organising maps (SOMs) to link accelerometery signatures with behavioural patterns in free-roaming terrestrial animals
Source: Sci Rep. 2021 Jun 30;11:13566. doi: 10.1038/s41598-021-92896-4 (PMC8245572; doi:10.1038/s41598-021-92896-4)
Supplement: Supplementary file 1 — Supplementary Informations. [file 41598_2021_92896_MOESM1_ESM.pdf]

SUPPLEMENTARY MATERIAL

Quantifying finer-scale behaviours using self-organising maps (SOMs) to link accelerometery signatures with behavioural patterns in free-roaming terrestrial animals.

Nicole Galea[a], Fern Murphy[a], Joshua L. Gaschk[a], David S. Schoeman[b],[c], Christofer J. Clemente[a,b]

a School of Science and Engineering, University of the Sunshine Coast, 90 Sippy Downs Drive, Sippy Downs QLD 4556.

b Global-Change Ecology Research Group, University of the Sunshine Coast, 90 Sippy Downs Drive, Sippy Downs QLD 4556.

c Centre for African Conservation Ecology, Department of Zoology, Nelson Mandela University, Port Elizabeth, South Africa.

Corresponding Author: C. J. Clemente

Email: cclement@usc.edu.au,

This supplementary material contains

Supplementary Tables 1-7

Supplementary Figures 1-3

Matlab interface instructions

Software is available via the link below.

https://figshare.com/articles/Galea_et_al_2019/9978797

Supplementary Table 1. Behaviour Matrix. Description of the 12-behaviours/activities, how each activity was defined in the MATLAB interface, and the activity numbers allocated to each.

Activity	Behaviour groups	Description of Movement	Activity Number
Lying	Sedentary	Inactive, flank horizontal or sternum.	1
Sitting	Sedentary	Inactive, sitting on hind-legs under the body, rump on the ground, forelegs out the front or held under body.	2
Grooming	Sedentary	Horizontal flank, rump or body horizontal on the ground with recurring head movements.	3
Watching	Sedentary	Inactive with four legs under body OR rump on the ground with head still and focusing on an object/toy or prey.	4
Eating	Eating and locomotive	Inactive, body in a standing or sitting position, head is lowered, jaw movement, purring white noise.	5
Walking	Eating and locomotive	Slow gait speed, symmetric diagonal gait	6
Trotting	Eating and locomotive	Two - beat gait, symmetric diagonal gait one foot is supporting the body, medium – high gait speed	7
Galloping	Hunting	Three – beat gait, fast gait speed, one set of legs on a diagonal move forward and land together whilst the other two legs advance forward and separately land.	8
Jumping	Hunting	All for legs leave the ground and landing with the forelegs first and one leg with s digital extensors extend the foot, then the other legs follow.	9
Pouncing	Hunting	All four feet leave the ground and land to extend forelegs out to capture prey/object under front of body.	10
Swatting	Hunting	Either forelegs extend to swipe in front or in the air.	11
Bite/Hold	Hunting	Forelegs hold the prey/object whilst the head and open jaw attach, some repeated biting, back legs can kick up against prey/object.	12

Supplementary Table 2. Predictor Variables. Descriptions of the predictor variables that were used to fit the model.

Parameter	Description
**Axes**	x (side to side), y (back and forth), z (up and down)
**Mean**	Mean, calculated over a moving window, of the chosen parameters.
**s.d.**	Measures of the spread of the signal for each axis or statistic.
**Signal Magnitude Area**	Measurement of movement activity within all three axes: SMA = $\frac{1}{N}(\sum_{i=1}^{N} x_i + \sum_{i=1}^{N} y_i + \sum_{i=1}^{N} z_i)$
**Overall dynamic body acceleration**	The absolute sum of DBA for each axis (x,y,z): OBDA = $DBA_x + DBA_y + DBA_z$
**Vectorial dynamic body acceleration**	VeDBA = $\sqrt{DBA\ x^2 + DBA\ y^2 + DBA\ z^2}$
**Skew**	Measure of the lack of symmetry of the distribution
**Kurtosis**	Measure of weight of the tails relative to a normal distribution.

Supplementary Table 3. SOM Accuracy. Model accuracy based upon a SOM trained with 20,000 random samples of BibOFF data to predict the remaining BibOFF data. Accuracy metrics are calculated as shown in Figure 2.

Activity	Overall Accuracy	Precision	Sensitivity	Specificity
Lying	0.996	0.999	0.93	1
Sitting	1	0.999	0.999	1
Grooming	0.997	0.378	0.358	0.998
Watching	0.999	1	0.993	1
Eating	0.997	0.989	0.834	1
Walking	0.989	0.927	0.998	0.988
Trotting	0.996	0.995	0.676	1
Galloping	0.995	0.998	0.964	1
Jumping	0.991	0.918	0.993	0.991
Pouncing	1	1	0.986	1
Swatting	0.998	0.993	0.994	0.999
Bite/Hold	0.996	0.996	0.981	0.999
Mean	**0.996**	**0.933**	**0.892**	**0.998**

Supplementary Table 4. SOM confusion matrix. Each row represents the predicted value in each behavioural group, while each column represents the instances observed for each behaviour. Numbers along the diagonal represent the true positives from the model.

	bite/ hold	eating	galloping	grooming	Juming	Lying	pouncing	sitting	swatting	trotting	walking	watching
bite/hold	9228	0	0	0	0	0	0	0	0	0	0	0
eating	0	24382	0	0	0	0	0	0	0	0	0	0
galloping	27	0	465	40	10	4	114	2	46	0	38	0
grooming	0	0	0	18316	0	0	0	0	0	0	0	0
jumping	0	0	0	0	2769	0	0	0	0	0	0	0
lying	348	12	1	5	0	25966	23	457	41	0	11	283
pouncing	132	0	60	12	464	48	2078	6	291	3	125	84
sitting	1	3	1	0	1	0	1	25579	1	0	2	0
swatting	0	0	0	0	0	0	0	0	17370	0	0	0
trotting	0	0	0	0	0	0	0	0	0	1054	0	0
walking	0	0	0	0	0	0	0	0	0	0	29639	0
watching	5	0	1	14	0	0	0	143	2	0	2	33079

Supplementary Table 5. Random forest Accuracy. Model accuracy based upon a SOM trained with 20,000 random samples of BibOFF data to predict the remaining BibOFF data. Accuracy metrics are calculated as shown in Figure 2.

Activity	Overall Accuracy	Precision	Sensitivity	Specificity
**Lying**	0.992	0.951	0.989	0.992
**Sitting**	0.986	0.944	0.952	0.991
**Grooming**	0.992	0.957	0.962	0.996
**Watching**	0.971	0.910	0.923	0.981
**Eating**	0.989	0.971	0.940	0.996
**Walking**	0.970	0.948	0.871	0.990
**Trotting**	0.999	0.764	0.979	0.999
**Galloping**	0.999	0.644	0.952	0.999
**Jumping**	0.995	0.736	0.944	0.996
**Pouncing**	0.997	0.782	0.907	0.997
**Swatting**	0.986	0.928	0.919	0.993
**Bite/Hold**	0.993	0.910	0.949	0.995
**Mean**	**0.989**	**0.871**	**0.941**	**0.994**

Supplementary Table 6. Random forest confusion matrix. Each row represents the predicted value in each behavioural group, while each column represents the instances observed for each behaviour. Numbers along the diagonal represent the true positives from the model.

	bite/hold	eating	galloping	grooming	jumping	lying	pouncing	sitting	swatting	trotting	walking	watching
bite/hold	8860	8	0	32	12	158	12	32	145	0	38	41
eating	122	23693	2	53	178	47	11	237	69	3	341	438
galloping	0	0	340	0	0	0	5	0	6	0	6	0
grooming	28	37	0	17602	0	201	37	117	23	0	74	179
jumping	0	26	3	0	2388	5	12	8	12	4	24	49
lying	74	0	0	33	15	24755	0	7	53	0	30	56
pouncing	18	0	22	1	42	16	1732	0	28	6	28	16
sitting	56	237	11	88	31	152	1	24727	82	0	176	405
swatting	323	0	15	12	102	170	154	68	16477	16	119	471
trotting	0	0	0	0	0	4	0	0	3	808	8	2
walking	183	180	92	381	352	318	210	518	399	210	28259	1345
watching	77	216	43	185	124	192	42	473	454	10	714	30444

Supplementary Table 7. Accelerometer data points. Table of total BiB_{OFF} and Bib_{ON} data points collected for each activity recorded.

Activity	BibOn	BibOff
Lying	16921	28748
Sitting	20854	28882
Grooming	11279	20252
Watching	38690	36900
Eating	21493	26928
Walking	33161	32967
Trotting	846	1153
Galloping	715	571
Jumping	2463	3588
Pouncing	3884	2452
Swatting	7280	19582
Bite/hold	2138	10766
Total	159724	212789

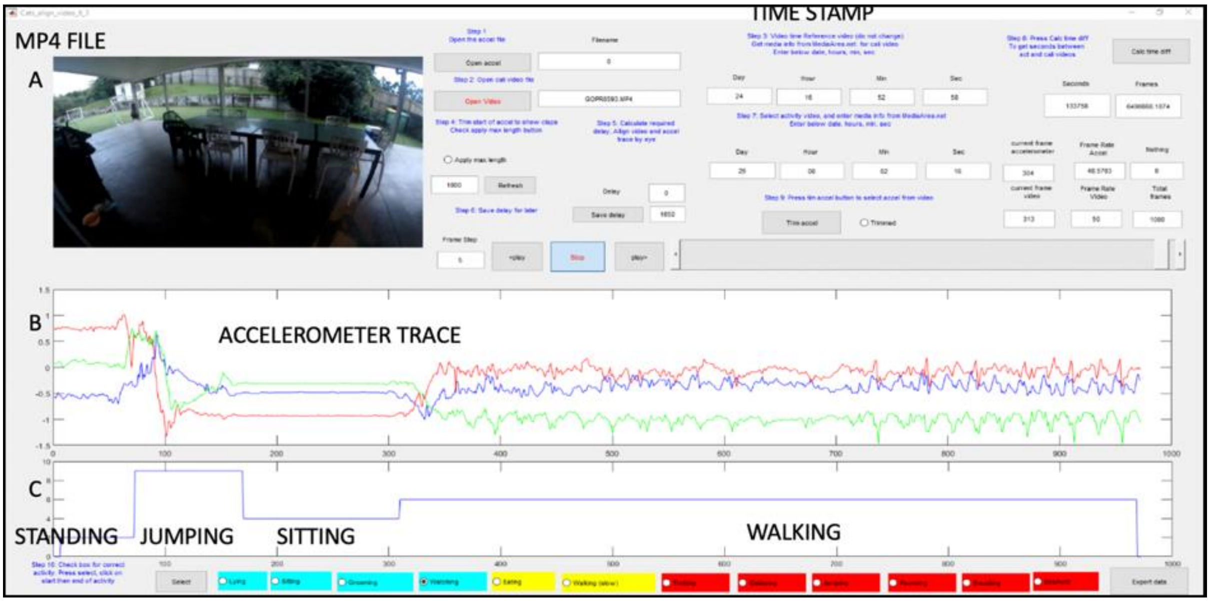

Supplementary Figure 1. Interactive purpose-built MATLAB interface. (**A**) the video window to watch the MP4 video files and each of the cat's movements. (**B**) Accelerometer trace window showing the; X axis-surge/Red; Y axis-sway/Green; Z axis-heave/Blue. (**C**) Activity allocation window, where each movement or behaviour was manually allocated from the trace window.

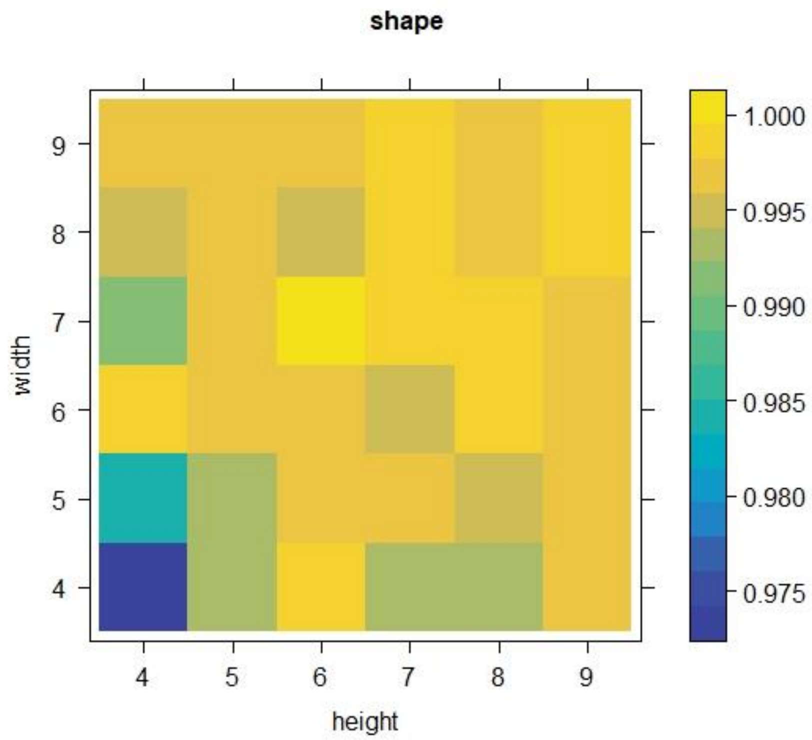

Supplementary Figure 2. Sensitivity analysis for the grid shape in the SOM. We varied the shape of the somgrid (n, m) in the supersom function in R e.g. supersom(TrainingData, grid = somgrid(n, m, “hexagonal")) from 4 to 9 units in length. We then tested each SOM using the testing dataset to create a confusion matrix and get an estimate of overall accuracy for each behaviour. The mean accuracy for all behaviours was then used to colour the heat map above. We selected the 7 x 7 SOM based on high accuracy and symmetry.

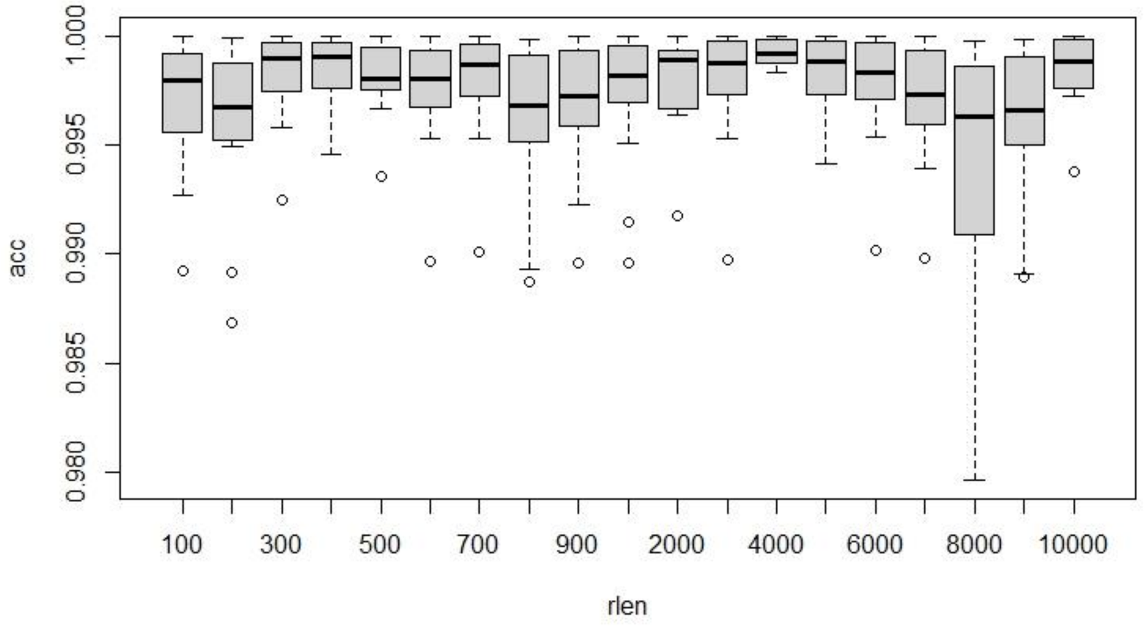

Supplementary Figure 3. Variation in the rlen argument in the supersom.R function. We varied the length of the rlen argument between 100 and 10000. The rlen argument indicates the number of times the data are presented to the network. We then tested each SOM using the testing dataset to create a confusion matrix and get an estimate of overall accuracy for each behaviour. The mean accuracy for all behaviours was then plotted at each rlen configuration. We found no evidence for an increase in accuracy with increasing rlen. Therefore we chose the default rlen value of 100 to reduce computational time.

1.1 MATLAB INTERFACE INSTRUCTION:

UPLOADING DATA -

1. Open the version file first (this will have the MATLAB code, fig, m and vline files) and will open the interface.
2. On the m. file click the RUN button on the top menu to Set a path
    a. Run _Set a path = this will apply the code to the fig file.

Step 1. Open Accel data = this will load the file date and time stamp. ex. 20-Nov-2018 15.05.53

Step 2. Open Video = this will load the file name. ex. GOPRO0001.MP4 – ensure to start with the first video so to calibrate the time stamps.

Step 3. Mediainfo = open the 'MediaInfo_GUI_18.08.1_Mac.dmg' file = open and click on the this this will go to the files (find the correct file and open and find the right video # – open). This will display the time stamp required for the Day:HR:Min:ss.
Apply the information from the time stamp into the relevant spots in Step 3. The 'DAY' is the number 1-14 days of the treatment – NOT the date.

Step 4. Max length Value = Apply a maximum length to the Accel trace to calibrate the first claps. ex. 1000.
Refresh = to trim the Accel. trace in the trace window. You can adjust the estimated maximum length and refresh to adjust to ensure you see the first 5 x claps.
>Play = to play the video to find the claps.

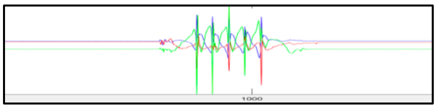

You can <Playback
Play the video to find the first claps and find the delay. This will have to be a visual estimation or guess, apply the estimated delay.

Step 5. Calculate the Delay time and Play back <> and forth to find the accurate delay to the trace data. This may require some adjustments and once the video mirrors the accelerometer. trace data it can be saved.

Step 6. Save Delay = this will be saved for later – play till the end of the trace and video, before uploading the second video.

Adding the following videos to the original cats accelerometer data.

Step 7. Mediainfo = open the ‘MediaInfo_GUI_18.08.1_Mac.dmg’ file = open and click on the this this will go to the files (find the correct file and open and find the right video # – open). This will display the time stamp required for the Day:HR:Min:ss. (change the information)
Apply the information from the time stamp into the relevant spots in Step 7. The ‘DAY’ is the number 2 or more days of the treatment – NOT the date.
Open Video = to upload the next video from the file.

Step 8. Calc time diff = This will set the time delays between the videos.

Step 9. Trim Accel **=** This will trim the accelerometer from the video. Check the Delay time as this may need adjusting again.

Step 10. Activity allocation = click on Activity (ex. sitting) then the Select and click (single) on the start of the activity and drag the + tool over the activity, then a single click on the end of the ex. sitting activity. The video and accelerometer trace should match up with the activity to show the activity. You will be able to distinguish the changes with the video. Manually find all activities and apply the step 10, repeat for all information in the trace data. – leave nothing un-labelled

Step 11. Export data = this will export to the file where the CSV and videos came from – and will be called the ex. project files\PROJECTS 2018\cat project 2018\GOPR0001_processed.txt - Now open in excel and SAVE

Repeat this process for all the video data until the trace data is complete.
